# Electrically-Responsive Reversible Polyketone/MWCNT Network through Diels-Alder Chemistry

**DOI:** 10.3390/polym10101076

**Published:** 2018-09-28

**Authors:** Rodrigo Araya-Hermosilla, Andrea Pucci, Patrizio Raffa, Dian Santosa, Paolo P. Pescarmona, Régis Y. N. Gengler, Petra Rudolf, Ignacio Moreno-Villoslada, Francesco Picchioni

**Affiliations:** 1 Programa Institucional de Fomento a la Investigación, Desarrollo e Innovación, Universidad Tecnológica Metropolitana, Ignacio Valdivieso 2409, P.O. Box 8940577, San Joaquín, Santiago 8940000, Chile; 2 Department of Chemistry and Industrial Chemistry, University of Pisa, Via Moruzzi 13, 56124 Pisa, Italy; andrea.pucci@unipi.it; 3 Department of Chemical Product Engineering, ENTEG, University of Groningen, Nijenborgh 4, 9747AG Groningen, The Netherlands; p.raffa@rug.nl (P.R.); d.s.santosa@rug.nl (D.S.); p.p.pescarmona@rug.nl (P.P.P.); 4 Zernike Institute for Advanced Materials, University of Groningen, Nijenborgh 4, 9747AG Groningen, The Netherlands; ryn.gengler@gmail.com (R.Y.N.G.); p.rudolf@rug.nl (P.R.); 5 Laboratorio de Polímeros, Instituto de Ciencias Químicas, Facultad de Ciencias, Universidad Austral de Chile, Valdivia 5110033, Chile; imorenovilloslada@uach.cl

**Keywords:** functionalised polyketone, MWCNT, electrically conductive plastic nanocomposite, reversible Diels-Alder, recycling, resistive heating annealing, reworkability

## Abstract

This study examines the preparation of electrically conductive polymer networks based on furan-functionalised polyketone (PK-Fu) doped with multi-walled carbon nanotubes (MWCNTs) and reversibly crosslinked with bis-maleimide (B-Ma) via Diels-Alder (DA) cycloaddition. Notably, the incorporation of 5 wt.% of MWCNTs results in an increased modulus of the material, and makes it thermally and electrically conductive. Analysis by X-ray photoelectron spectroscopy indicates that MWCNTs, due to their diene/dienophile character, covalently interact with the matrix via DA reaction, leading to effective interfacial adhesion between the components. Raman spectroscopy points to a more effective graphitic ordering of MWCNTs after reaction with PK-Fu and B-Ma. After crosslinking the obtained composite via the DA reaction, the softening point (tan(δ) in dynamic mechanical analysis measurements) increases up to 155 °C, as compared to the value of 130 °C for the PK-Fu crosslinked with B-Ma and that of 140 °C for the PK-Fu/B-Ma/MWCNT nanocomposite before resistive heating (responsible for crosslinking). After grinding the composite, compression moulding (150 °C/40 bar) activates the retro-DA process that disrupts the network, allowing it to be reshaped as a thermoplastic. A subsequent process of annealing via resistive heating demonstrates the possibility of reconnecting the decoupled DA linkages, thus providing the PK networks with the same thermal, mechanical, and electrical properties as the crosslinked pristine systems.

## 1. Introduction

Conductive polymer nanocomposites are an important class of materials used in a broad range of applications, including supercapacitors, solar cells, conductive adhesives, advanced electronic devices, among many others [1]. Such nanocomposites based on the direct mixing between carbon nanotubes (CNTs) and thermoplastic polymers often resulted in materials with outstanding performance due to the synergistic effect between the inherent features of CNTs and the type of interaction at the interface with the matrix [2]. The interface stability for CNT-reinforced thermoplastics is difficult to achieve due to the lack of effective interfacial adhesion between the components. As a consequence, for instance, thermoplastics display poor dispersion of CNTs during the melt state mixing process, thus resulting in poor exfoliation and aggregate structures [3]. New approaches, such as in-situ chemical and electrochemical polymerisation have substantially improved the effective exfoliation of entangled agglomerates of CNT bundles and ropes in thermoplastic matrices [4]. The latest progress in the field established that the dispersion, exfoliation, and stabilisation of CNTs are enhanced when an effective interfacial covalent bond is formed between filler and matrix [5]. Accordingly, the most common approach consists of grafting reactive moieties to the edges and sidewalls of CNTs, which then covalently interact with the polymer matrix [6,7]. However, this method presents disadvantages regarding the chemical oxidation of the sp^2^ graphitic arrangement of CNTs, thus diminishing their conductivity and mechanical performance [8,9]. An emerging alternative to the above problems is the direct mixing of CNTs with thermosetting resins that crosslink during conventional liquid processing. The employed chemical routes barely disrupt the graphitic structure of CNTs upon curing into solid structures [10,11]. CNT-polymer crosslinked nanocomposites offer many superior properties compared to thermoplastics-based ones, such as mechanical strength, dimensional stability at elevated temperature, solvent resistance, and reliable electric performance owing to the conductive network of well-dispersed CNTs [5]. Unfortunately, crosslinked polymer nanocomposites are generally not re-processable after their service life, thus hindering their recyclability in the “cradle to cradle” approach, a keystone in the current efforts for sustainability.

A promising strategy currently employed in the fabrication of re-workable and re-processable crosslinked polymer systems is the incorporation of thermally reversible moieties used for reprocessing (and recycling) the network after the product’s life ends [12]. The most common approach is based on Diels-Alder (DA) chemistry (reversible covalent cycloaddition) [13,14,15]. Mainly, crack propagation in DA crosslinked polymers is more likely to take place at the DA linkages because DA covalent bonds (given their shear sensitive character) are weaker than regular covalent bonds [16]. After grinding, thermal mending procedures provide the energy to activate the functional moieties and the process of reshaping and reconnection of linkages [17,18,19]. As a next step, thermal annealing is commonly performed to improve the structural properties of the material [20,21]. The latter further promotes the intrinsic ability of thermally reversible polymers to re-connect local areas with fast kinetics. For these mechanisms to come into play, DA active groups have to be present along the backbone, and this often requires long and cumbersome polymerisations and/or modification strategies. An easier alternative is to introduce alternating aliphatic polyketones (PK) by copolymerisation of carbon monoxide, ethylene, and propylene via the Paal-Knorr reaction to insert furan groups directly attached to the backbone chain [20,22,23]. This chemical reaction proceeds in the bulk with high yields and relatively fast kinetics, producing water as the only by-product [24]. The grafted furan groups allow for the formation of a three-dimensional polymer network after crosslinking with aromatic bis-maleimide. The material indeed formed a thermally reversible and self-healing thermoset by means of a DA and retro-DA (r-DA) sequence employing conventional heating procedures and post-curing thermal annealing [20]. The combination of such materials with CNTs should then merge the advantages of thermosetting nanocomposites (see above) with the self-healing and recycling possibilities of this easily affordable (and thus industrially attractive) resin.

Herein we report on an intrinsic (thermally reversible) crosslinked conductive nanocomposite that displays thermomechanical re-workability, stable electrical response, annealing by resistive heating, and which behaves like a thermoplastic upon heating/pressing for recycling. This electrically conductive nanocomposite stems from the chemical modification of an alternating aliphatic polyketone grafted with furan groups (PK-Fu) via the Paal-Knorr reaction, crosslinked with B-Ma and reinforced with multi-walled carbon nanotubes (MWCNTs) via reversible Diels-Alder cycloaddition. Spectroscopic tools were employed to study the modification of the starting polyketone, the crosslinking of the resulting polymer, and the reversible chemical bonding with the nanofiller via the DA and retro-DA sequences. Thermomechanical tests allowed evaluating the mechanical performance, re-workability, and recyclability of the composite. The results concerning the material’s modulus and impedance measurements before and after recycling were contrasted with further electrical resistive heating annealing procedures in order to simultaneously improve the mechanical and electrical performance of the nanocomposite. The intrinsic chemical and resistive heating responses were studied by attenuated total reflection Fourier transform infrared (ATR-FTIR) spectroscopy and IR thermography, respectively. The morphology of the system and dispersion/exfoliation of MWCNTs were analysed by electronic microscopy before and after annealing by resistive heating.

## 2. Materials and Methods

The alternating aliphatic polyketone (PK) was synthesised according to Mul et al. [25]. The resulting co- and ter-polymer of carbon monoxide presents a total olefin content of 30% of ethylene and 70% of propylene (PK30, MW 2687 Da). Furfurylamine (Fu, Sigma Aldrich, ≥99%, Zwijndrecht, The Netherlands) was freshly distilled before use, and benzylamine (Bea) (Sigma Aldrich 99%), MWCNTs (O.D. 6–9 nm, average length 5 µm, Sigma Aldrich 95% carbon), DMSO-*d*_6_ (Laboratory-Scan, 99.5%, The Netherlands), (1,1-(methylenedi-4,1-phenylene)bis-maleimide (B-Ma, Sigma Aldrich 95%), 1-Methyl-2-pyrrolidone (NMP, Sigma Aldrich, 99.5%), Dimethylformamide (DMF, Sigma Aldrich 99.8%), tetrahydrofuran (THF, Laboratory-Scan, 99.5%), chloroform (CHCl_3_, Laboratory-Scan, 99.5%) and deuterated chloroform (CDCl_3_, Sigma Aldrich 99.8 atom% D) were purchased and used as received.

### 2.1. Functionalisation of Polyketone with Furan and Benzyl Groups

**Figure 1:**
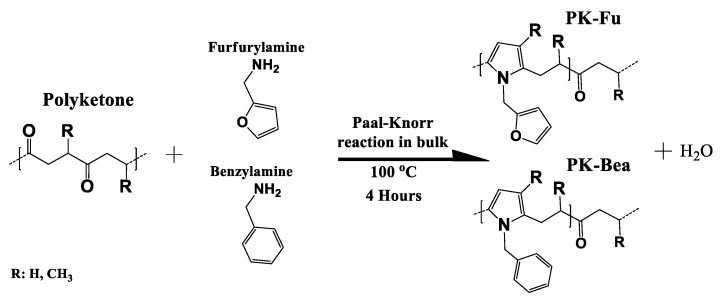
Schematic representation of PK functionalised with furan (PK-Fu) or benzyl (PK-Bea) groups via Paal-Knorr reaction.

The reaction between PK and furfurylamine was carried out in bulk by the Paal-Knorr reaction, and the molar ratio between the reactants (i.e., the di-carbonyl group of polyketone and the amine group of furfurylamine) was established as percentages with a maximal conversion of 80% according to Zhang et al. and Toncelli et al. [20,21]. In order to establish more clearly the role of the furan motifs, a polymer grafted with benzyl groups was prepared by the Paal-Knorr reaction and used as a reference. This compound, called PK-Bea hereafter, displays the same backbone structure as PK-Fu, but has non-reactive pendant groups, at least in our conditions, via Diels-Alder cycloaddition (Figure 1, see SI1 for experimental procedures).

### 2.2. Functionalisation of MWCNTs with PK-Fu or B-Ma via Diels-Alder Reaction

PK-Fu (0.95 g) was dissolved in 5 mL of NMP, and added to 0.05 g of MWCNTs (previously sonicated for 30 min in 5 mL of NMP). The reaction was set under vigorous stirring at 50 °C for 24 h using an oil bath equipped with a temperature controller. The reaction mixture was repeatedly washed with THF and filtered in order to recover the MWCNTs grafted with PK-Fu (see product recovered in Table S2A). The remaining solvent was removed under vacuum at 50 °C for 48 h. The same procedure was used to functionalise MWCNTs with bis-maleimide. Likewise, MWCNTs and PK-Bea were mixed following the same protocol to generate a reference sample (Table S2A and S2B).

### 2.3. Preparation of PK-Fu/B-Ma/MWCNT Composite

The thermoset nanocomposite was prepared by one-pot solvent-mix containing equimolar amounts of PK-Fu and B-Ma (at a furan/maleimide ratio of 1:1) and 5 wt.% of MWCNTs. The reactants were previously dissolved in THF (≈10 wt.%) and bath sonicated for 30 min in a 150 mL round-bottomed flask equipped with a magnetic stirrer. The reaction mixture was heated to 50 °C for 24 h to form the crosslinked network under reflux. After reaction, the solvent was removed under vacuum at 50 °C overnight. The resulting powder was divided into small portions of ≈500 mg that were moulded into rectangular bars at 150 °C for 30 min under a pressure of 40 bar. A neat thermoset sample without MWCNTs was also prepared for comparison. After moulding, the samples were cooled down to room temperature (30 min) and then stored at −17 °C for further analysis. 

### 2.4. Characterisation

The elemental composition of the samples was analysed using a Euro EA elemental analyser (Langenselbold, Germany). ^1^H NMR spectra were recorded on a Varian Mercury Plus 400 MHz apparatus (Agilent, Santa Clara, CA, USA) using deuterated chloroform as solvent. FT-IR spectra were collected using a Perkin-Elmer Spectrum 2000 (San Francisco, CA, USA). Sample pellets were prepared by mixing potassium bromide (KBr) with the polymer (≈1.5 wt.%). The powder was then kept under vacuum at 50 °C for 24 h to remove residual water. ATR-FTIR spectra were recorded using a Thermo Nicolet NEXUS 670 FTIR (Waltham, MA, USA). Differential Scanning Calorimetry (DSC) analysis was performed on a TA-Instrument DSC 2920 (Eschborn, Germany) under N_2_ atmosphere. The samples were weighed (10–17 mg) in an aluminium pan, which was then sealed. Then, the samples were heated from 0 °C or 40 °C to 180 °C and then cooled to 0 or 40 °C. Four heating-cooling cycles were performed at a rate of 10 °C/min. Gel Permeation Chromatography (GPC) measurements were performed with a HP1100 Hewlett-Packard (Wilmington, Philadelphia, PA, USA). The equipment consists of three 300 × 7.5 mm PLgel 3 µm MIXED-E columns in series and a GBC LC 1240 RI detector (Dandenong, Victoria, Australia). The samples were dissolved in THF (1 mg/mL) and eluted at a flow rate of 1 mL/min and a pressure of 100–140 bar. The calibration curve was made using polystyrene as standard and the data were interpolated using the PSS WinGPC software. Dynamic Mechanical Thermal Analyses (DMTA) were conducted on a rheometrics scientific solid analyser (RSA II) (TA Instruments, New Castle, DE, USA) under air environment using the dual cantilever mode at an oscillation frequency of 1 Hz and a heating rate of 3 °C/min. The samples for DMTA analysis were prepared by compression moulding of 500 mg of the composite into rectangular bars (6 mm wide, 1 mm thick, 54 mm long) at 150 °C for 30 min under a pressure of 40 bar to ensure full homogeneity. Resistive heating annealing was performed on the rectangular bars used for DMTA analysis. The setup consisted of a power supply (EA-PS 3150-04 B) and a multimeter (FLUKE 175). Electrical parameters were measured on samples connected to a conventional circuit using copper clamps holders. Thermal images of the samples subjected to electrical current were obtained using a FLUKE IR thermometer camera (VT02) (Everett, WA, USA). Impedance analysis was performed with a KEYSIGHT E4990A (Santa Rosa, CA, USA) impedance analyser in the frequency ranges from 100 to 30 MHz at room temperature. Samples with both sides displaying an area of 0.019 mm^2^ and 1 mm thick were connected directly to the electrodes for testing. Raman spectra were recorded on an Alpha 300 (Witec) (Ulm, Germany) using a laser at 532 nm; the spectral range from 1000 to 3500 cm^−1^ was investigated. At least 3 different points were analysed to reliably describe the bulk of each sample regarding possible structural inhomogeneities. The acquisition time was 50 s and 10 spectra were accumulated to reduce the signal to noise ratio. X-ray photoelectron spectroscopy (XPS) was carried out with a SSX-100 (Surface Science Instruments) (Mountain View, CA, USA) spectrometer equipped with a monochromatic Al Kα X-ray source (hν = 1486.6 eV) that operates at a base pressure of 3 × 10^−10^ mbar. The energy resolution was set at 1.45 eV, the photoelectron take-off angle was 37° with respect to the surface normal, and the diameter of the analysed spot was 600 μm. Spectra were collected at a minimum of two different spots on each sample, checked for consistency, and averaged for each spectral region. The data were fitted using the Winspec software (developed at the L.I.S.E. of the University of Namur, Belgium), applying the Shirley background and a linear combination of Gaussian and Lorentzian peaks. All samples were re-suspended in toluene and deposited dropwise on Au substrates. After the solvent was evaporated, the samples were transferred into ultra-high vacuum via a load-lock system. Samples containing the pure polymer display broadened peaks due to sample charging effects. Therefore, the fitting width was set at 2.1 eV. The binding energy scale was corrected by using the C=C sp^2^ signal at 284.4 eV as a reference. Scanning electron microscope micrographs were taken with a Philips XL30S Environmental SEM-FEG instrument (Eindhoven, The Netherlands). High-resolution images were acquired on freshly broken surfaces.

## 3. Results and Discussion

### 3.1. PK Functionalised with Furan and Benzyl Groups via Paal-Knorr Reaction

The solvent-free Paal-Knorr reaction between PK and the amine compounds was carried out according to different molar ratios between the 1,4-di-carbonyl groups of polyketone and furfurylamine or benzylamine aiming at a maximal conversion of 80% (Figure 1).

The summary of the experimental results is displayed in Table 1.

**Figure 2:**
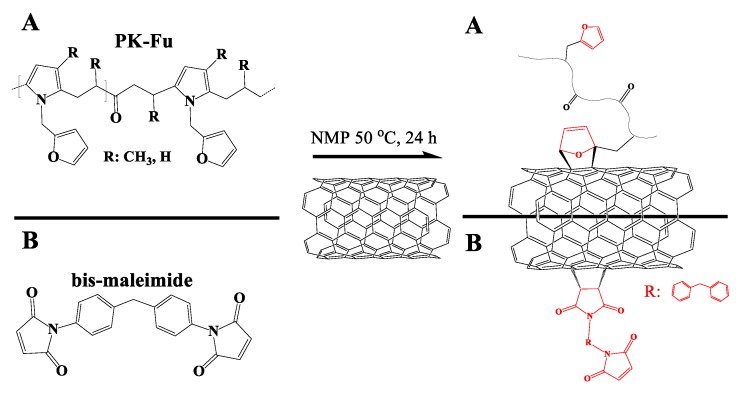
Functionalisation of MWCNTs with (**A**) PK-Fu (diene) and (**B**) bis-maleimide (dienophile) via Diels-Alder reaction.

Notably, functionalised polyketones display a carbonyl conversion (Cco, measured by elemental analysis) close to the target conversion (efficiency, η > 90%, see Table 1). This result validates the robustness and versatility of the Paal-Knorr reaction with polyketones [23]. For brevity, the spectral characterisation of PK-Fu is displayed in SI1B, which shows the ^1^H NMR spectrum of the polymer mixed with furfurylamine before and after the chemical reaction. After modification, the characteristic peaks of the pyrrole appear in the spectrum, namely the proton signal ascribed to CH_2_ groups between the pyrrole and the furan groups at 4.9 ppm, the one related to the pyrrole group at 5.8 ppm and proton signals of the furan moieties at 5.9, 6.2 and 7.3 ppm [20,23]. The FT-IR spectrum of the same sample is displayed in Figure 2B. It is possible to notice the appearance of C-H stretching related to the heterocyclic groups around 3150–3115 cm^−1^ (pyrrole and furan groups), the C=O stretching of the residual carbonyl groups at 1707 cm^−1^, the C=C stretching of the heterocyclic groups at 1507 cm^−1^, the pyrrole C–N stretching at 1345 cm^−1^, the furan C–O–C stretching at 1073 cm^−1^, and eventually, the out-of-plane bending of the furan ring C–H bonds at 735 cm^−1^.

The T_g_ of the functionalised polymers was measured by DSC (Table 1). As expected, the chemical modification of the di-carbonyl arrangement into pyrrole groups increases the rigidity of the backbone. Indeed, PK-Fu and PK-Bea show a T_g_ of 31 °C and 42 °C, respectively, i.e., much higher than that of PK before modification (−12 °C). GPC measurements showed no significant differences in the PDI of the two systems, indicating the absence of significant irreversible side reactions. The comparison of the two systems suggests that benzyl pendant groups confer stronger supramolecular interaction among the macromolecular chains with respect to that provided by furan moieties. This highlights the versatility of the PK system, whose modification with different amino compounds grafted on the same backbone allows tuning the T_g_ value of the ultimate polymer.

### 3.2. Functionalisation of MWCNTs with PK-Fu or B-Ma via Diels-Alder Reaction and PK-Bea via Physical Interactions

Figure 2 displays schematically the reaction between MWCNTs and PK-Fu or B-Ma (Table S2A). After functionalisation, the MWCNTs were repeatedly washed with THF in order to remove the unreacted PK-Fu, PK-Bea and B-Ma while the amount of the grafted component was evaluated by elemental analysis (EA) (Table S2B).

The results obtained clearly establish the presence of nitrogen in all the functionalised MWCNT samples, whereas only traces (0.01 wt.%) were found for pristine MWCNTs. The values of nitrogen measured for the grafted MWCNT/PK-Fu showed the highest N content close to 34 wt.%, whereas this drops to about 8 wt.% for MWCNT/B-Ma accordingly. In the case of MWCNTs mixed with PK-Bea, no covalent interaction is expected; thus, the N content of 13 wt.% arises from physical interactions between PK-Bea and the filler, due to π-π stacking [26,27]. The results clearly suggest a strong chemical interaction between MWCNTs and PK-Fu different to a physical π-π stacking observed in the case of MWCNT/PK-Bea.

**Figure 3:**
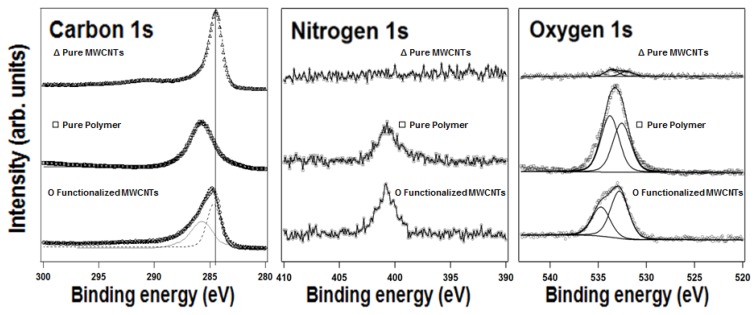
X-ray photoemission spectra of the C1s, N1s and O1s core level regions of MWCNTs functionalised with PK-Fu. Data and fitting lines are shown.

In literature, the formation of covalent bonds between cyclopentadiene, furan and bis-maleimide with CNTs via DA chemistry has been already proved by means of X-ray photoelectron spectroscopy (XPS) [28,29]. In connection with those findings, XPS analysis of the MWCNTs functionalised with PK-Fu clearly confirmed the effectiveness of the DA grafting mechanism (Figure 3).

It is worth noting that in the carbon 1s spectra, differences between the modified and un-modified MWCNTs surfaces can be identified; for the un-modified MWCNTs, the main peak at 284.5 eV, accompanied by the shake-up feature at about 6.0 eV higher binding energy characteristic of π-π conjugated systems, perfectly fits with C sp^2^ reported in the literature [28,29]. On the other hand, the C1s spectrum of the pure polymer shows a broad feature peaked at 286.0 eV and resulting from three components, i.e. C–C sp^2^, –CH_2_ –CH_3_ sp^3^ and CH_2_–C–CH_3_, which are too closely spaced in binding energy [29] to be resolved in our experimental conditions. The spectrum for the pure polymer also shows O1s components attributed to O=C at 532.4 eV and to O–C at 533.8 eV, as well as a N1s peak at 401.5 eV. All these are in remarkable agreement with the PK-Fu structure. For the product obtained after the grafting PK-Fu to the MWCNTs, the presence of the polymer is evident from the N1s and O1s intensities, but can be also clearly observed in the carbon spectra, namely by the presence of a broad peak, which was fitted here with a linear combination of the pure polymer and MWCNT signal (respectively in dotted and dashed line). While it is difficult to conclude from the C1s spectrum that the sidewall functionalisation of MWCNTs with PK-Fu proceeds via C–C bonding, in the O1s spectrum the O-C component results shifted by 1 eV to higher binding energy, clearly pointing at a charge redistribution resulting from the furan attachment on the MWCNT surface.

**Figure 4:**
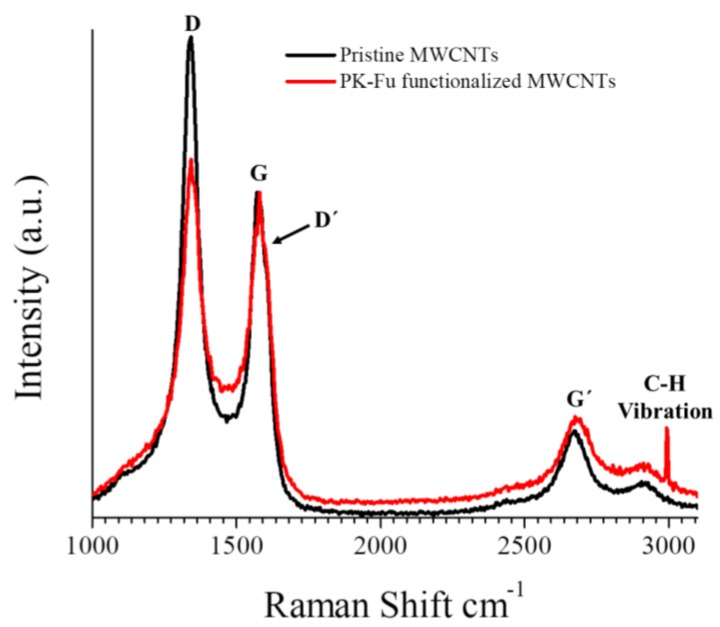
Raman spectra (normalised at the G band, 1580 cm^−1^) of pristine and PK-Fu-functionalised MWCNTs.

The Raman spectra of pristine and PK-Fu-functionalised MWCNTs are displayed in Figure 4. 

For brevity, only few spectra are displayed (see supporting information Figure S3 for PK-Bea and B-Ma functionalised MWCNTs). It is widely known that Raman spectroscopy provides useful information regarding the degree of crystallinity, lattice defects, and graphitic ordering of MWCNTs. As shown in Figure 4, the G band, which testifies to the graphitic crystalline arrangement (1580 cm^−1^), the D band related to defects (1345 cm^−1^) and the D-band overtone, G’ band, indicative of the multi-walled structure (2684 cm^−1^) are detected. The band centred at 2995 cm^−1^ (C-H vibration of alkanes) is only detected in the presence of the polymer. The intensity ratio between D and G bands is also a useful standard measurement of the amount of defects in carbon nanotubes [30]. Accordingly, the D/G ratio decreases from 1.49 for pristine MWNTs to 1.29 for PK-Fu functionalised MWCNTs. This suggests that after the cycloaddition step subsequent rehybridisation restores the sp^2^ state, recovering the aromaticity of the system in the defect region of MWCNTs, as recently reported [31]. Highly defective graphene layers can also be followed by the key analysis of intensity ratio between G’ and D bands, that increases from 0.17 for pristine MWCNTs to 0.27 for the less entangled PK-Fu functionalised MWCNTs (see Figure S4). In summary, even though PK-Fu modifies the graphitic surface of MWCNTs, its presence possibly improves the long-range graphitic order of pristine MWCNTs.

**Figure 5:**
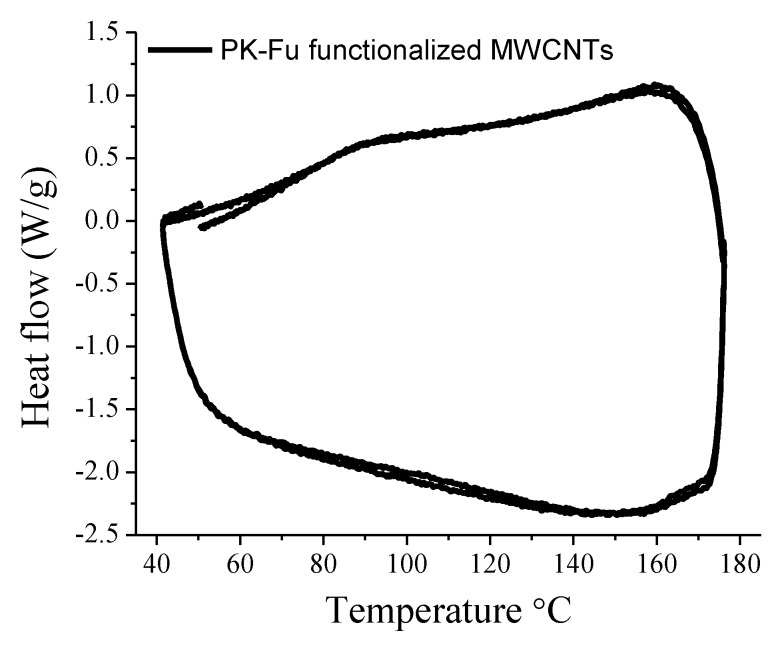
DCS thermal cycles of the PK-Fu functionalised MWCNTs.

DSC analysis of the PK-Fu nanocomposite (Figure 5) evidences two endothermal transitions: one around 75–80 °C, associated with the glass transition of the material, and the second from 120 to 170 °C, centred at 160 °C and associated to the retro-DA reaction. The energy required for the retro-DA process reaches 11.6 J/g, a value that perfectly correlates with the energy associated to retro-DA reaction for conventional thermally reversible furane/maleimide crosslinked systems (see below) [21,32]. Overall, the MWCNT/PK-Fu represents a consistent example of reversible grafting between a graphitic electrically-conductive material and a polymer matrix; new investigations are now in progress to modulate the grafting extent through the choice of the processing parameters. 

### 3.3. PK-Fu/B-Ma/MWCNT Composite

**Figure 6:**
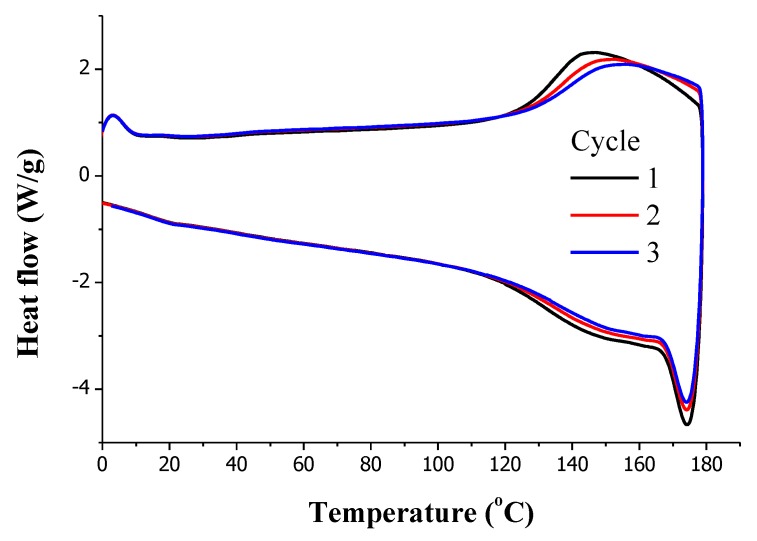
DSC thermal cycles of PK-Fu crosslinked with B-Ma and reinforced with MWCNTs.

The MWCNT/PK-Fu nanocomposite was further crosslinked with B-Ma via Diels-Alder reversible reaction aiming at the production of electrically conductive soft and chemically reversible polymer networks. The crosslinked nanocomposite was then obtained in the form of highly homogeneous rectangular bars by one-pot solvent-mix containing equimolar amounts of PK-Fu and B-Ma (ratio 1:1 between Fu and Ma groups) and 5 wt.% of MWCNTs followed by compression moulding. The neat thermoset and the nanocomposite were characterised by DSC (Figure S5 and Figure 6, respectively) to study their thermal behaviour, reversibility, and the exo-/endo-thermal processes related to the DA and retro-DA sequence respectively. The thermograms display a broad endothermic transition in the range of temperature between 120 °C and 180 °C for each consecutive thermal cycle. The similarity between the three thermal cycles clearly indicates the re-workable character of the crosslinked PK-Fu, even when reinforced with the MWCNTs [20,21]. The endothermic transition corresponds to the r-DA process. Therefore, the peak of the curves corresponds to the average temperature at which the majority of the DA adducts are broken, and the area under the curve associated to this peak is related to the energy absorbed during the cleavage of the DA adducts.

The DSC analysis of the nanocomposite shows that the presence of MWCNTs has no apparent effect on the thermal behaviour of the thermoset [22]. Indeed, the energy associated to the r-DA process was calculated from the DSC heating trace to be 11.1 J/g for the nanocomposite and 10.9 J/g for the neat thermoset, thus suggesting that the presence of the filler does not interfere with the r-DA process of the matrix. Namely, even if a covalent interaction between matrix and filler might occur via the DA mechanism, the corresponding thermal transition is not visible in the DSC traces, due to either overlap with the one associated to adducts of Fu and Ma, or simply relatively low concentration.

**Figure 7:**
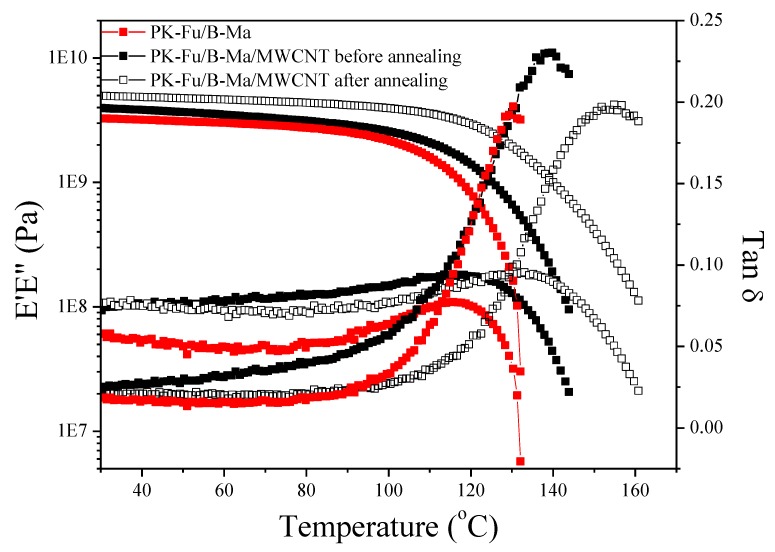
DMTA of PK-Fu crosslinked with b-Ma (PK-Fu/B-Ma, filled red squares) and PK-Fu crosslinked with B-Ma and reinforced with 5 wt.% of MWCNTs before (PK-Fu/B-Ma/MWCNT, filled black squares) and after resistive heating annealing (PK-Fu/B-Ma/MWCNT, black empty squares) for 24 h using 35 V.

The thermomechanical features of PK-Fu crosslinked with B-Ma and reinforced with MWCNTs were also determined after compression a grinded sample at 150 °C and 40 bar for 30 min. This process was supposed to favour r-DA mechanism and the consequent de-crosslinking of the thermoset nanocomposite. The latter aims at building a proof of concept related to the reversibility of crosslinked systems as high performance conductive and recyclable thermoplastics. DMTA analysis performed on rectangular bars of this material (obtained after compression moulding) shows that the filler leads to clear enhancements of softening point (tan(δ) peak), loss and elastic moduli with respect to the neat crosslinked system (Figure 7).

Notably, the softening point (tan(δ)) is increased from ~130 °C for the PK-Fu crosslinked with B-Ma to ~140 °C for the PK-Fu/B-Ma/MWCNT nanocomposite, which, after resistive heating, increased to ~155 °C. This can be explained by the fact that MWCNTs reinforce the structure of the matrix, which consequently displays increased rigidity. This effect is aided by the effective interfacial interactions between the matrix and the filler via DA cycloaddition, as suggested by XPS and Raman analyses. As a result, the filler helps in dispersing the applied force, and consequently improves the mechanical performance of the composite. Another phenomenon that possibly contributes to the softening point enhancement is the potential catalytic effect exerted by MWCNTs on the DA reaction between PK-Fu and B-Ma adducts, which leads to a higher crosslinking density upon resistive heating [33].

**Figure 8:**
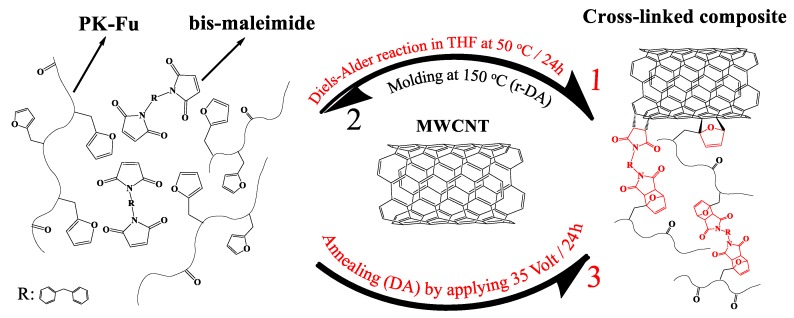
Schematic representation of PK-Fu crosslinked with B-Ma and reinforced with MWCNTs. Arrows display the process of: (1) crosslinking (DA), (2) moulding (partial r-DA) and (3) annealing (DA) by resistive heating.

**Figure 9:**
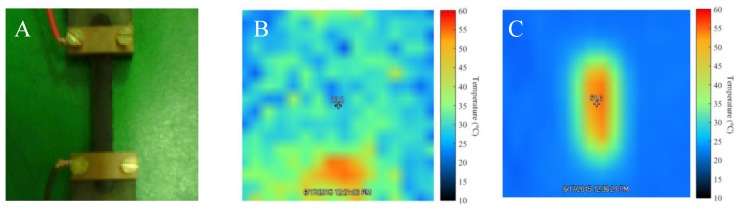
(**A**) Photograph of the PK-Fu/B-Ma/MWCNT composite sample with 5 wt.% of MWCNTs connected to the electrical circuit and its thermal images before (**B**) and during the application of a voltage of 35 V (**C**).

It is worth noting that the elastic modulus (E’) and the softening temperature (tan(δ)) are significantly improved after resistive heating for 24 h at 35 V, as compared with the system after moulding. The electrical current effectively induces heat dissipation from MWCNTs, which triggers the reconnection between decoupled DA adducts, and thus, re-establishes the crosslinked network [34]. A schematic representation of the proposed mechanism is reported in Figure 8. In order to confirm this idea, we monitored the temperature of the sample during the application of the voltage (Figure 9).

Notably, the well-distributed red colour all along the film surface (Figure 9C) clearly suggests the homogeneous distribution of MWCNTs in the crosslinked nanocomposite. The temperature reached by the network during the application of the electrical current corresponds to about 55–60 °C, i.e., a temperature at which the direct DA reaction is predominant even if not exclusively present [20,35,36]. Although higher temperatures might be possible by using a different filler load or higher voltage, the equilibrium nature of the DA reaction (i.e., both direct and retro-DA taking place) might be still observed at these temperatures, thus possibly resulting in relatively low temperature self-healing behaviour.

The sequence of crosslinking in solution, de-crosslinking during moulding, and annealing by resistive heating of the nanocomposite was also monitored by ATR-FTIR spectroscopy (Figure S6). The normalised spectra at 2930 cm^−1^ (C–H stretch, Figure S6) and at 1145 cm^−1^ (C–N stretching band [37], Figure S6B) evidence the intensity variations of the C–N–C peak at 1185 cm^−1^ attributed to the stretching of the succinimide ring in the DA adduct. It is worth noting that the DA band decreased in intensity after moulding (decoupling of DA adducts, red curve), along with the appearance of furan and maleimide moieties (736 cm^−1^ and 672 cm^−1^, respectively), whereas it promptly recovered after annealing (blue curve) to intensities similarly collected from the nanocomposite thermally crosslinked in solution (black curve).

**Figure 10:**
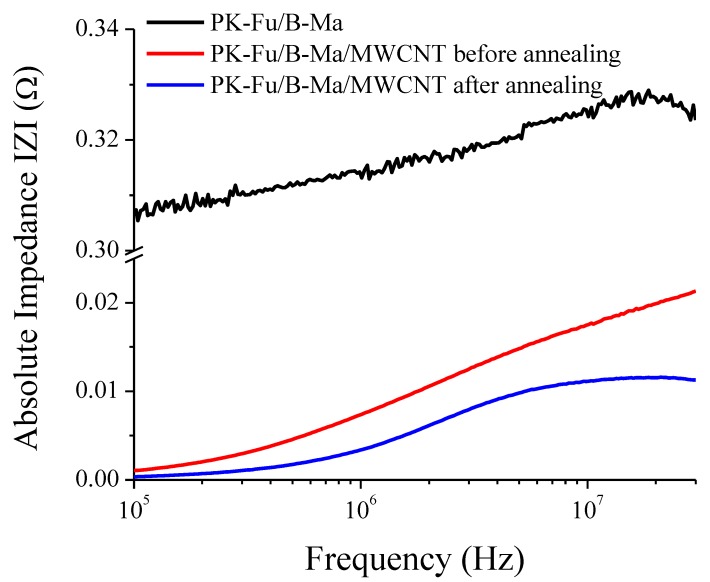
Variation of absolute impedance of PK-Fu crosslinked with B-Ma (PK-Fu/B-Ma) and of the crosslinked PK-Fu/B-Ma/MWCNT crosslink composite before and after resistive heating annealing using 35 V for 24 h.

**Figure 11:**
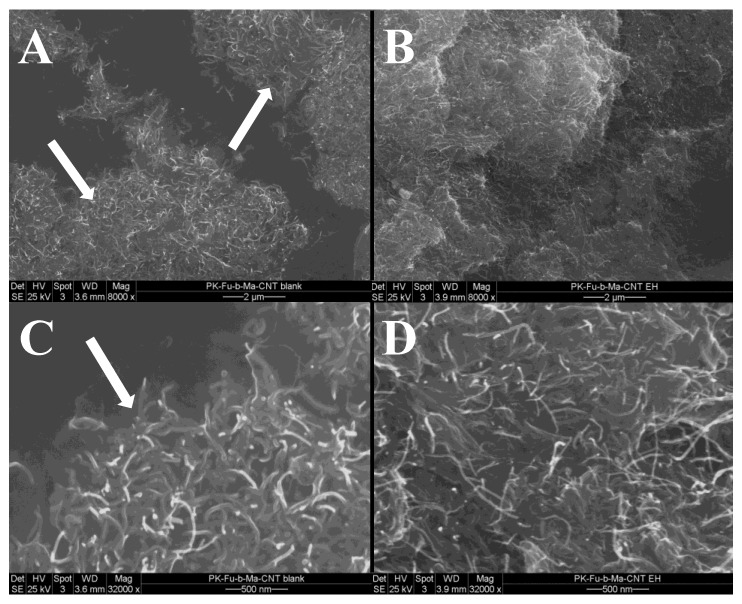
SEM micrographs of PK-Fu/B-Ma/MWCNTs reversible crosslinked composite after moulding (**A**,**C**) and after annealing by resistive heating (**B**,**D**).

According to the results displayed in Figure S6, part of the DA adducts are broken after moulding, which could possibly favour re-agglomeration of MWCNTs, thus leading to poor conductivity. To support this hypothesis, impedance measurements and morphological studies by SEM were combined in order to figure out the degree of dispersion of MWCNTs in the matrix (Figure 10 and Figure 11).

Figure 10 indicates that the impedance (i.e., the total resistance of the material) of the PK-Fu/B-Ma/MWCNT crosslinked system decreases one order of magnitude as compared to the un-reinforced system. Even more remarkable is the fact that the material reduces its impedance by half and becomes more electrically conductive upon annealing (compare red and blue curves in Figure 10), as is clearly noticed for frequencies >5 MHz, when the impedance becomes nearly constant. This can be due to the effective interfacial adhesion between the components and good dispersion of the MWCNTs that also suggests less matrix/filler mobility upon different frequencies [38,39]. 

Contrast mode SEM micrographs [40] corroborate the results obtained from electrical measurements on the same sample after being moulded and successively annealed by resistive heating (Figure 11). On the freshly broken surfaces of the composite after moulding (Figure 11A,C), bundles of MWCNTs (indicated by white arrows) can be observed. Conversely, after resistive heating (Figure 11B,D) MWCNTs are better distributed, thus confirming the ability of the electric current to restore a more effective percolative network. The re-distribution of the MWCNTs in the crosslinked matrix is also evidenced by the average outer diameter analysis of the MWCNTs before and after annealing (Figure S7). The analysis of the diameter average of the graphitic assemblies reported in the SEM micrograph suggests an effective modification of the MWCNT distribution within the polymer matrix after the annealing procedure (Figure S7). During resistive heating, the increased polymer mobility possibly favours the redistribution of macromolecular chains, thus increasing the MWCNT dispersion (average diameter passed from about 15 to about 11 nm) aided by the better interfacial adhesion between the components.

## 4. Conclusions

We have demonstrated that furan-functionalised polyketone (PK-Fu) becomes electrically conductive through addition of moderate amounts of MWCNTs (5 wt.%), and that, in this way, intrinsic self-healing can be promoted by thermally reversible Diels-Alder crosslinks activated by resistive heating. Spectroscopy investigations showed that MWCNTs contribute in the reversible crosslinking of the PK-Fu with B-Ma due to the diene/dienophile character of the MWCNT and their increased graphitic order after network formation. In addition, electronic microscopy investigations revealed the effective distribution of MWCNTs. By means of the presented approach, it was possible to realise a crosslinked nanocomposite displaying a relatively high softening point (tan(δ)) at 155 °C, i.e., about 25 °C higher than that without MWCNTs. After grinding the electrically conductive PK network, compression moulding at 150 °C activates the retro-DA process, thus making it possible to reshape the nanocomposite as thermoplastic. A subsequent process of annealing via resistive heating at 35 V for 24 h favoured the reconnection of the decoupled DA linkages, and provided the crosslinked PK nanocomposite with the same thermal, mechanical, and electrical characteristics as the undamaged polymer network. 

In summary, we believe that our results may pave the way towards the cost-effective industrial production of electrically-conductive crosslinked and recyclable nanocomposites endowed with intrinsic re-connection features triggered by resistive heating activation of reversible Diels-Alder adducts. 

## Figures and Tables

**Table 1:** Experimental results of PK modified with furfurylamine (PK-Fu) and benzylamine (PK-Bea).

Run	C_CO_ (%) ^a^	η (%) ^b^	T_g_ (°C) ^c^	PDI ^d^
**PK-Fu**	74	92	31	2.3
**PK-Bea**	73	91	42	2.2

^a^ Carbonyl conversion; ^b^ conversion efficiency; ^c^ glass transition temperature ^d^ polydispersity index.

## Supplementary Materials

The following are available online at http://www.mdpi.com/2073-4360/10/10/1076/s1, Table S1: Synthesis of PK-Fu and PK-Bea, Table S2: Calculation of grafted MWCNTs with PK-Fu, PK-Bea and bis-maleimide, Figure S3: Raman spectra of pristine and PK-Fu, B-Ma, PK-Bea functionalized MWCNTs, Figure S4: SEM micrographs of (A) pristine MWCNTs and (B) PK-Fu functionalized MWCNTs, Figure S5: DSC thermal cycles of crosslinked PK-Fu with B-Ma, Figure S6: ATR-FTIR spectra of PK-Fu crosslinked with B-Ma and reinforced with MWCNTs, Figure S7: Statistical analysis of PK-Fu/B-Ma/MWCNT micrographs.
