# Supplementary material for: Electrically-Responsive Reversible Polyketone/MWCNT Network through Diels-Alder Chemistry
Source: Polymers (Basel). 2018 Sep 28;10(10):1076. doi: 10.3390/polym10101076 (PMC6403874; doi:10.3390/polym10101076)
Supplement: Supplementary file 1 [file polymers-10-01076-s001.pdf]

Supporting information

Electrically-responsive reversible polyketone/MWCNT network through Diels-Alder chemistry

Rodrigo Araya-Hermosilla [1,*], Andrea Pucci [2], Patrizio Raffa [3], Dian Santosa [3], Paolo P. Pescarmona [3], Régis Y. N. Gengler [4], Petra Rudolf [4], Ignacio Moreno-Villoslada [5] and Francesco Picchioni [3,*]

[1] Programa Institucional de Fomento a la Investigación, Desarrollo e Innovación, Universidad Tecnológica Metropolitana, Ignacio Valdivieso 2409, P.O. Box 8940577, San Joaquín, Santiago, Chile.
[2] Department of Chemistry and Industrial Chemistry, University of Pisa, Via Moruzzi 13, 56124 Pisa, Italy; andrea.pucci@unipi.it
[3] Department of Chemical Engineering/Product technology, ENTEG, University of Groningen, Nijenborgh 4, 9747AG Groningen, The Netherlands; d.s.santosa@rug.nl (D.S); p.raffa@rug.nl (P.R); p.p.pescarmona@rug.nl (P.P.P.).
[4] Zernike Institute for Advanced Materials, University of Groningen, Nijenborgh 4, 9747AG Groningen, The Netherlands; p.rudolf@rug.nl (Petra R.); ryn.gengler@gmail.com (R.Y.N.G.)
[5] Laboratorio de Polímeros, Instituto de Ciencias Químicas, Facultad de Ciencias, Universidad Austral de Chile, Chile; imorenovilloslada@uach.cl (I.M.)
* Correspondence: f.picchioni@rug.nl (F.P.); Tel.: +31-50-3634333; rodrigo.araya@utem.cl (R.A.-H.); Tel.:+56-2-27877911

S1. Synthesis of PK-Fu and PK-Bea

Table S1A. Experimental conditions and results of PK modified with furfurylamine and benzylamine

Run	**Ratio NH_2/C=O**	***Amine compound (g)***	***M_w^y (g/mol)[a]***	***N (g)[b]***	***EA (N%)[c]***
**PK-Fu**	0.8	11.8	192.6	1.602	5.84
**PK-Bea**	0.8	13.01	216.6	1.512	5.28

[a] Mw of the pyrrolic functionalized unit, [b] grams of nitrogen in the product, [c] percentage of nitrogen according to elemental analysis.

The synthesis of PK-Fu and PK-Bea was carried out in a sealed 250 mL round-bottom glass reactor with a reflux condenser, a U-type anchor impeller, and an oil bath for heating. After 10 g of PK was preheated to a liquid state at 100 °C, furfurylamine or benzylamine was added dropwise to the reactor during the first 20

min. The stirring speed was set at 600 rpm and the reaction time was fixed to 4 h. Initially, the reaction mixture was colorless but then changed to brown because of pyrrole formation on the polymer backbone. The resulting polymers were washed 3 times with Milli-Q water to remove any unreacted furfurylamine or benzylamine. Thereafter, the remaining water was removed under vacuum in a freeze dryer for 72 h.

The carbonyl conversion (C_{co}) can be calculated by:

$$C_{co} = \frac{y}{y+x} \cdot 100\% \quad (1)$$

where x and y represent the moles of di-ketone and pyrrolic units after conversion, respectively, in a definite mass of product (g_{prod}). y can be calculated as follows:

$$y = \frac{wt(N)}{A_m\ (N)} \quad (2)$$

where wt(N) represents the weight in grams of nitrogen in g_{prod} according to elemental analysis, and A_m(N) is the atomic mass of nitrogen. x can be calculated as follows:

$$x = \frac{g_{prod} - y \cdot M_w^{\ y}}{M_w^{\ pk}} \quad (3)$$

where $M_w{}^{y}$ represents the molecular weight of the pyrrolic functionalized unit and $M_w{}^{pk}$ the molecular weight of the di-ketone unit (131.6 g/mol). The conversion efficiency η can be defined as the ratio between the carbonyl conversion C_{co} according to the moles of dicarbonyl in the feed $C_{co}{}^{feed}$:

$$\eta = \frac{C_{co}}{C_{co}{}^{feed}} \cdot 100 \quad (4)$$

The $C_{co}{}^{feed}$ is calculated as follows:

$$C_{co}{}^{feed} = \frac{Mol_{amine}}{Mol_{PK30}} \cdot 100 \quad (5)$$

with Mol_{amine} representing the moles of amine compounds and Mol_{PK30} the moles of di-carbonyl units in the feed.

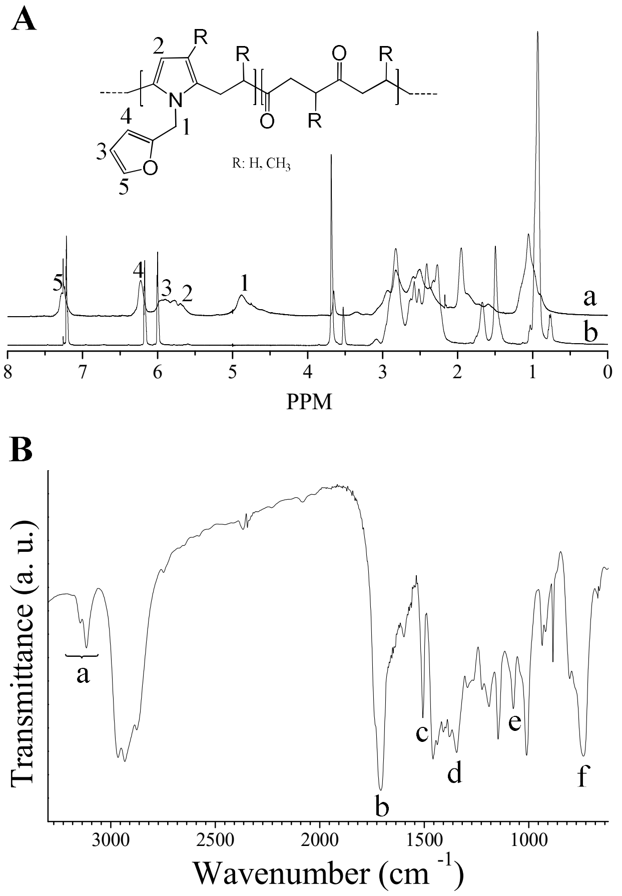

Figure S1B. A) ^{1}H NMR spectra of PK before (b) and after chemical modification with furfurylamine (a). B) FT-IR spectra of PK after chemical modification with furfurylamine a) νs C-H heterocyclic groups at 3150-3115 cm^{-1}, b) νs C=O at 1707 cm^{-1}, c) νs C=C at 1507 cm^{-1}, d) νs N-C at 1345 cm^{-1}, e) νs C-O-C at 1073 cm^{-1}, f) cyclic out-of-plane C-H bending at 735 cm^{-1}.

S2. Calculation of grafted MWCNTs with PK-Fu, PK-Bea and bis-maleimide.

Table S2A Experimental conditions for functionalization of MWCNTs with PK-Fu, PK-Bea and bis-maleimide.

Run	Polymer (g)	b-Ma (g)	MWCNT (vol%)	Mixture (g)	Product recovered (g)
[a] **MWCNT/PK-Fu**	0.95	---	5	1	0.077
[b] **MWCNT/PK-Bea**	0.95	---	5	1	0.055
[c] **MWCNT/B-Ma**	---	0.95	5	1	0.0504

Table S2B Experimental results for the functionalization of MWCNTs with PK-Fu, PK-Bea and bis-maleimide.

Run	N content (%)	Grafted (%)[a]
**MWCNT/PK-Fu**	2.45	34
**MWCNT/PK-Bea**	0.83	13
**MWCNT/B-Ma**	0.62	8

Grafted compounds (%) on the surface of MWCNTs are estimated by [a] elemental analysis (nitrogen (**N**) content).

The percentage of grafted product (grafted (%)) can be calculated as follows:

$$\text{grafted}(\%) = \frac{\text{Prod}_{\text{recov}}}{\text{Graf}_{\text{prod}}} \cdot 100 \qquad (1)$$

where $\text{Prod}_{\text{recov}}$ represents the amount of material recovered after filtering the excess of compound that did not react with MWCNTs; $\text{Graf}_{\text{prod}}$ represents the amount of PK-Fu, PK-Bea or b-Ma (in grams) grafted on the MWCNTs surface. The $\text{Graf}_{\text{prod}}$ is calculated as follows:

$$\text{Grafprod} = \text{Mw}^{\text{y}} \cdot \text{moles (N)} \qquad (2)$$

where M_w^y represents the molecular weight of the functionalized pyrrolic unit of PK-Fu and PK-Bea (in the case of B-Ma, the two nitrogens of the molecule are considered) and moles (N) are the moles of the pyrrolic unit or maleimide groups according to the moles of nitrogen obtained by elemental analysis (see molecular structures in Figures 1 and 3 of the main text). The moles of nitrogen are calculated as follows:

$$\mathrm{moles(N)} = \frac{\mathrm{g(N)}}{\mathrm{A_m(N)}} \tag{3}$$

where $\mathrm{g(N)}$ represents the weight in grams of nitrogen according to the elemental analysis and $A_m(N)$ the atomic mass of nitrogen. Finally, the $\mathrm{g(N)}$ can be calculated as follows:

$$\mathrm{g(N)} = \mathrm{Prod_{recov}} \cdot \mathrm{N_{content}} \tag{4}$$

where $\mathrm{N_{content}}$ represents the percentage of nitrogen estimated by elemental analysis.

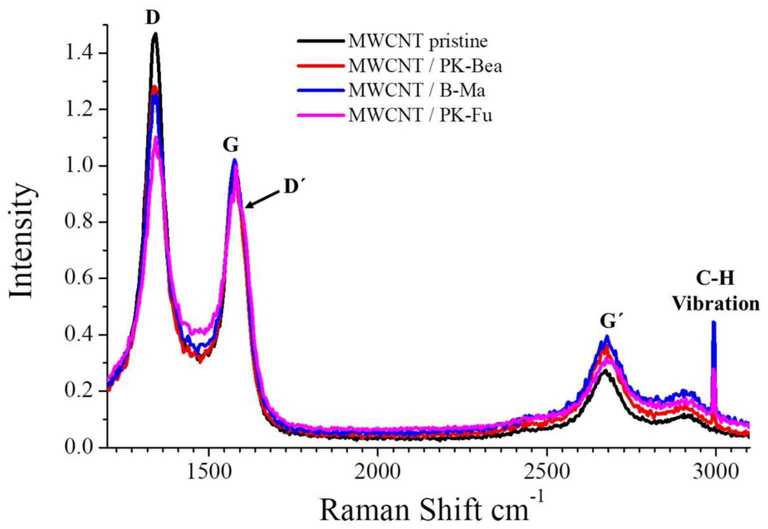

Figure S3. Normalized Raman spectra (at the G band, 1580 cm^{-1}) of pristine and PK-Fu, B-Ma, PK-Bea-functionalized MWCNTs. Pristine MWCNTs are used as reference.

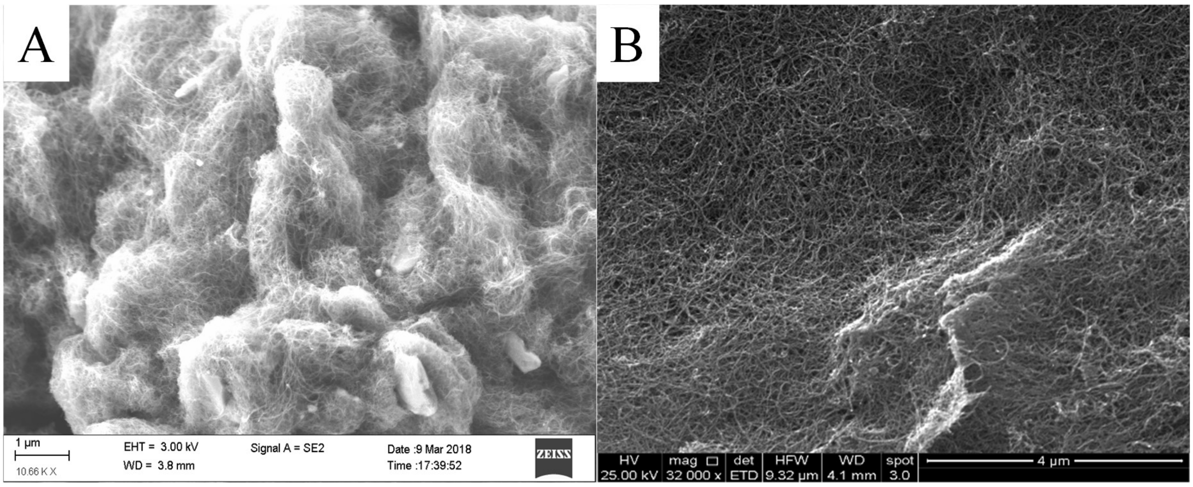

Figure S4. SEM micrographs of A) pristine MWCNTs and B) PK-Fu functionalized MWCNTs.

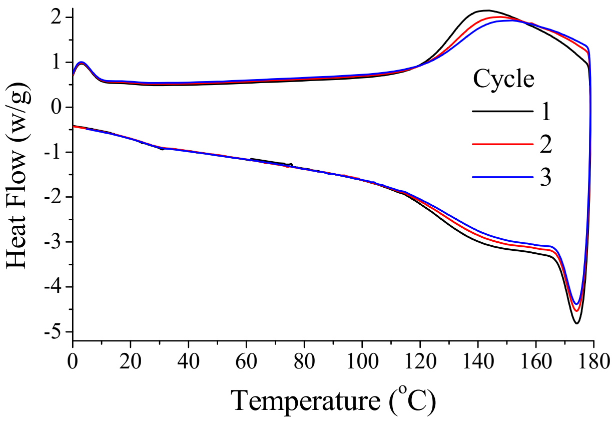

Figure S5. DSC thermal cycles of crosslinked PK-Fu with B-Ma. Ratio Fu / Ma = 1.

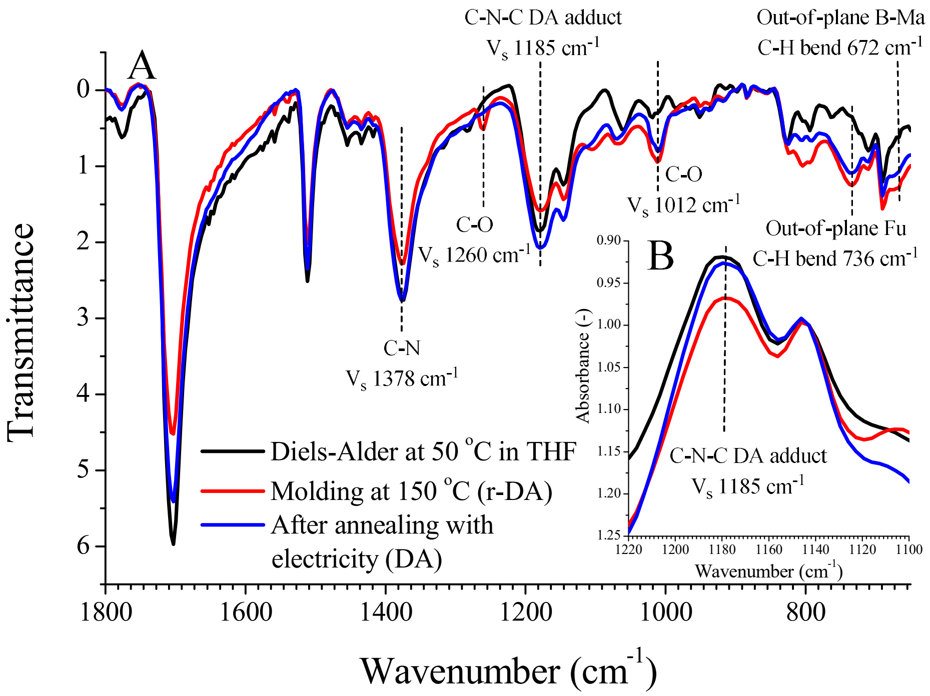

Figure S6. ATR-FTIR spectra of PK-Fu crosslinked with B-Ma and reinforced with MWCNTs. The different colours refer to the processes of: crosslinking (DA) (black), moulding (partial rupture of DA adducts or r-DA) (red) and annealing by resistive heating (reconnection of DA adducts) (blue)

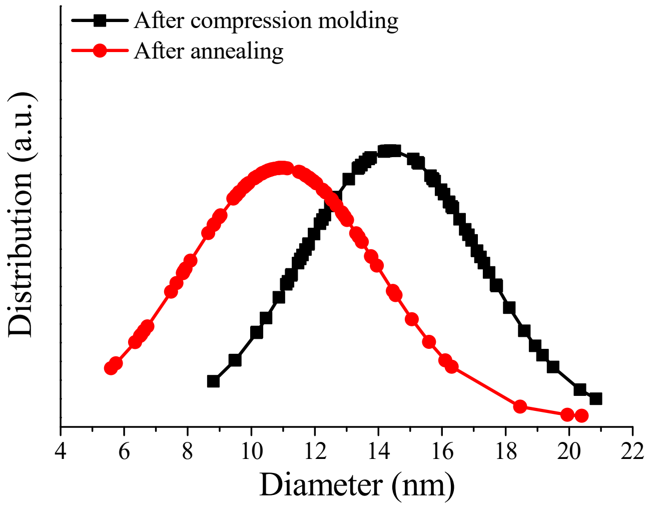

Figure S7. Statistical analysis of PK-Fu/B-Ma/MWCNTs micrographs of reversible crosslinked composite after moulding (black curve) and after annealing by resistive heating (red curve).
